# *Ex Vivo* Effect of Varespladib on Secretory Phospholipase A2 Alveolar Activity in Infants with ARDS

**DOI:** 10.1371/journal.pone.0047066

**Published:** 2012-10-11

**Authors:** Daniele De Luca, Angelo Minucci, Marco Piastra, Paola E. Cogo, Francesca Vendittelli, Laura Marzano, Leonarda Gentile, Bruno Giardina, Giorgio Conti, Ettore D. Capoluongo

**Affiliations:** 1 Laboratory of Clinical Molecular Biology, Department of Biochemistry, University Hospital “A.Gemelli”, Catholic University of the Sacred Heart, Rome, Italy; 2 Pediatric Intensive Care Unit, Department of Anaesthesiology and Intensive Care, University Hospital “A.Gemelli”, Catholic University of the Sacred Heart, Rome, Italy; 3 Pediatric Intensive Care Unit, Department of Pediatrics, Hospital and University of Padua, Padua, Italy; Ospedale Pediatrico Bambino Gesu', Italy

## Abstract

**Background:**

Secretory phospholipase A2 (sPLA2) plays a pivotal role in acute respiratory distress syndrome (ARDS). This enzyme seems an interesting target to reduce surfactant catabolism and lung tissue inflammation. Varespladib is a specifically designed indolic sPLA2 inhibitor, which has shown promising results in animals and adults. No specific data in pediatric ARDS patients are yet available.

**Methods:**

We studied varespladib in broncho-alveolar lavage (BAL) fluids obtained *ex vivo* from pediatric ARDS patients. Clinical data and worst gas exchange values during the ARDS course were recorded. Samples were treated with saline or 10–40–100 µM varespladib and incubated at 37°C. Total sPLA2 activity was measured by non-radioactive method. BAL samples were subjected to western blotting to identify the main sPLA isotypes with different sensitivity to varespladib. Results was corrected for lavage dilution using the serum-to-BAL urea ratio and for varespladib absorbance.

**Results:**

Varespladib reduces sPLA2 activity (*p*<0.0001) at 10,40 and 100 µM; both sPLA2 activity reduction and its ratio to total proteins significantly raise with increasing varespladib concentrations (*p*<0.001). IC_50_ was 80 µM. Western blotting revealed the presence of sPLA2-IIA and –IB isotypes in BAL samples. Significant correlations exist between the sPLA2 activity reduction/proteins ratio and PaO_2_ (rho = 0.63;*p*<0.001), PaO_2_/FiO_2_ (rho = 0.7; *p*<0.001), oxygenation (rho = −0.6; *p*<0.001) and ventilation (rho = −0.4;*p* = 0.038) indexes.

**Conclusions:**

Varespladib significantly inhibits sPLA2 in BAL of infants affected by post-neonatal ARDS. Inhibition seems to be inversely related to the severity of gas exchange impairment.

## Introduction

Secretory phospholipases A2 (sPLA2; phosphatide 2-acylhydrolase, EC 3.1.1.4) constitute a group of enzymes responsible for surfactant phospholipids catabolism and release of inflammatory mediators [1], [2]. sPLA2 are involved in the physiopathology of acute respiratory distress syndrome (ARDS): in fact, sPLA2 activity is increased in the lung of affected patients [3] and positively correlates with the predicted mortality and the degree of lung tissue inflammation [4], [5], [6]. sPLA2 levels correlate negatively with surfactant activity and compliance, as well [2], [7], [8]. Recently, we reported similar findings in pediatric ARDS: sPLA2 correlates with the clinical severity, the degree of ventilatory support and the gas exchange impairment [9].

A wide body of literature suggests that sPLA2 would be a potential target to treat ARDS, because its inhibition might block the vicious cycle linking surfactant degradation and lung inflammation [10]. While neonatal respiratory distress syndrome (iRDS) is efficaciously treated with exogenous surfactant, the usefulness of such therapy in ARDS beyond the neonatal age, is not yet well established [11]. Conversely, since the sPLA2-induced surfactant dysfunction is a typical feature of ARDS, the enzymatic inhibition could spare the endogenous pool or protect the exogenously administered surfactant [6], [10].

Varespladib sodium (also known as A-001, LY315920, S-5920) is a potent indole-based sPLA2 inhibitor that was optimized by structure-based drug design to inhibit some pulmonary isotypes of this enzyme (especially sPLA2-IIA [12]), while it is less potent against pancreatic sPLA2-IB and has no effect on cytosolic phospholipases [13], [14]. Nowadays, this is the sPLA2 inhibitor with more available pharmacologic data: varespladib has been intravenously administered in adults with septic shock [15], [16] and is presently under advanced clinical investigation for sickle cell disease-induced acute chest syndrome (IMPACTS trial, NCT00434473) [17]. Varespladib methyl is the oral pro-drug of varespladib sodium and it has been studied for chronic and acute cardiovascular disease in two trials (PLASMA [18], FRANCIS [19]).

Up to now, no specific data are available on pediatric ARDS patients: the aim of the present study is to investigate the effect of varespladib in broncho-alveolar lavage fluids obtained *ex vivo* from small infants suffering from ARDS.

## Materials and Methods

### Subjects

This study was conducted in two academic pediatric intensive care units (PICU) in a six months period. Eligible babies were all infants after the first 30 days and ≤1 year of age, who met the definition of ARDS according to the American-European Consensus criteria [20]. Exclusion criteria were: 1) lung congenital malformations; 2) need for thoracic surgery; 3) extremely critical conditions impeding the broncho-alveolar lavage (BAL) procedure; 4) patients on extra-corporeal life support. The study protocol and consent form were approved by institutional review boards of Universities of Rome and Padua; written informed consent was obtained from parents or guardians before the enrolment.

### Broncho-alveolar lavage and clinical data collection

Non-bronchoscopic BAL [21] was performed within 6 h from the fulfilling of ARDS criteria. Since this is a part of our routine protocol for microbiological surveillance, no procedure was performed solely for the study purposes and no change was provided to the routine clinical assistance. As previously published [9], BAL was performed according to the advices of the European Respiratory Society Pediatric Task Force [21]. In details, two sequential and separate aliquots of 1 mL/kg 0.9% NaCl warmed at 37°C were instilled; each instillation was followed by three respiratory cycles. A straight, snub-nosed, end-hole suction catheter was gently advanced into the endotracheal tube, while continuing ventilation through a Y-connector. When resistance was met, suctioning with 50 mmHg of negative pressure was applied. The procedure was performed with the infant's head turned 90° to the left and then to the right and repeated twice. The first fluid aspirated reflecting bronchial *milieu*, was sent for microbiological culture. The remaining fluid was diluted with 0.9% saline up to 5 mL and centrifuged at 3000 rpm for 10′. Cell-free supernatants were separated and immediately frozen at −80°C. Samples were excluded from further analysis if there was visible blood contamination.

Clinical history was recorded and broncho-pulmonary dysplasia (BPD) was diagnosed according to the United States National Institute for Child Health and Human Development definition [22]. All infants were ventilated using Servo-I ventilator (Maquet Critical Care, Solna, Sweden) in pressure controlled modality, allowing 5–6 mL/kg tidal volume. All babies have an indwelling arterial line, as per our clinical monitoring policy. We took data from our computerized bedside monitoring systems where vital parameters, mechanical ventilation data and blood gas values are recorded every 8 hours for mechanically ventilated babies, as per our internal protocol. The worst blood gas and pH values, lowest arterial oxygen saturation (SatO_2_), highest mean airway pressure (Pāw) and inspired oxygen fraction (FiO_2_) during the ARDS course were recorded. Lowest PaO_2_/FiO_2_ ratio was considered, as well. Oxygenation index (OI) and a modified ventilatory index (VI) [23] were calculated as follow: OI = [Pāw ^x^ FiO_2_/PaO_2_]; VI = [respiratory rate **^x^** (Pāw) **^x^** PaCO_2_/1000]. Moreover, within 6 hours from the ARDS diagnosis, single breath static respiratory system compliance was measured during a passive exhalation, after adequate end-inspiratory occlusion. Compliance was included into the “Murray's lung injury score modified for children” to assess the severity of ARDS at the enrolment [24].

### Laboratory methods

BAL specimens were thawed only once for the experiments, within six months from the collection and were subdivided in 4 aliquots in which sPLA2 activity was measured after the addition of 10, 40 or 100 µM varespladib or an equal volume of 0.9% saline. sPLA2 total activity was measured using a high-sensitivity non-radioactive commercially available kit (Assay Designs, Ann Arbor, MI, USA), which uses hexadecanoylthio-1-ethylphosphorylcholineas substrate. Details of this method have been described elsewhere [25]. Varespladib or normal saline were added just after the reaction buffer and samples were incubated at 37°C for 30′ before the addition of the substrate. Coefficient of variation was always ≤4%, while intra-assay and inter-assay variability were <5% and <9%, respectively. Varespladib was liberally provided by Anthera Pharmaceuticals as a powder of sodium-varespladib (A-001, Anthera Pharmaceuticals, Hayward, CA, USA) and it was diluted in bi-distilled water at convenient concentrations. Since varespladib solutions had a slightly yellow color, results were corrected for the varespladib absorbance.

Urea and proteins were also measured in BAL fluids, as previously described [9], [26]; Serum urea values obtained during the routine clinical tests in the same day of BAL procedure was used to calculate the serum-to-BAL urea ratio. Total phospholipid concentration was measured by analysis of lipid phosphorous, as previously described [27]. Coefficient of variation for these assays was always <5%. All measurements were performed in triplicate, by investigators blinded for the infants' clinical data.

The presence of the main sPLA2 subtypes (sPLA2-IIA and sPLA2-IB) that are differently targeted by varespladib, was also investigated by western blotting. In detail, BAL samples were lyophilized and 20 µg of BAL proteins were re-suspended in phosphate-buffered saline and dye-sample loading buffer (50 mM Tris–HCl, pH 6.7; 10% glycerol; 3% SDS; 1% β-mercaptoethanol; 0.01% bromophenol blue). These solutions were boiled at 100°C for 10′, centrifuged at 450 g for 5′ at 4°C and then used for each blot. Electrophoresis was performed using 12% SDS polyacrilamide and then sPLA2-IIA monoclonal antibody (Cayman Chemical, Ann Arbor, MI, USA) or sPLA2-IB polyclonal antibody (Santa-Cruz Biotechnology, Santa Cruz, CA, USA) specific for human phospholipases were incubated for 2 h (dilution 1∶200). The primary antibody was removed and immune-reactive bands were incubated with a peroxidase-conjugated secondary antibodies (at 1∶2000 dilution in blocking solution) for 1 h at room temperature. Human recombinant sPLA2IB and –IIA were used as controls. Blotted proteins were revealed using Immobilon Western Chemiluminescent HRP Substrate (Millipore, Billerica, MA, USA) according with manufacturer's protocol with maximum exposure time of 1′.

### Statistics and calculations

Normal distribution of data was primarily verified with Shapiro-Wilk test and median (interquartile range) or mean ± standard deviation were used, as appropriate. sPLA2 activity was uncorrected when comparing aliquots of the same BAL specimen. Other analyses were performed correcting for total protein content or serum-to-BAL urea ratio, as multiplying coefficient to transform BAL into epithelial lining fluid (ELF) concentrations [26].

Data were analysed using Friedman and Wilcoxon tests (for *post-hoc* comparisons). *Ex vivo* IC_50_ value, that is the sPLA2 remnant activity, was determined, taking into consideration sPLA2 activity reductions which were calculated as follows:
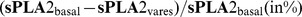
In this formula, sPLA2_basal_ is the enzyme activity in basal conditions (with addition of saline) and sPLA2_vares_ is the activity measured after the addition of increasing drug concentrations. Spearman correlation was finally performed. Statistical analyses were done using SPSS for Windows rel.15.0 (SPSS Inc., Chicago, IL, USA) and *p*-values<0.05 were considered to be statistically significant.

## Results

During the study period ten PICU-admitted babies were considered eligible for the study and all were enrolled. Table 1 reports basic clinical data of our population. All babies survived and were successfully discharged from the PICUs. On average, 3 ± 0.5 mL of fluid was aspirated with the non-bronchoscopic BAL procedure (on average 60% of the instilled volume). Total proteins and phospholipids in BAL fluids were 206 (161–290) mg/dL and 30.6±9.6 µg/mL, respectively. All BAL samples were sterile at the microbiological culture and did not show visible blood contamination.

**Table 1:** Clinical data.

Infants	10
Age (months)	3.5 (2–6)
Weight (kg)	5 (4–8)
GA (weeks)	36 (30–39)
Male sex	6 (60)
Prematurity	5 (50)
OI	15 (9.2–21)
PaO_2_/FiO_2_	95.8 (77.7–107.8)
VI	24 (13.5–67.5)
Murray's score	12.5 (11.7–14.5)
Duration of mechanical ventilation (days)	13.5 (7.7–17.2)
ARDS causes	5 H1N1 flu, 3 RSV bronchiolitis, 1 pneumonia, 1 severe sepsis
Co-morbidities	3 BPD, 2 Down syndrome

Data are expressed as median (i.q. range) or number (%). GA: gestational age at the birth; OI: oxygenation index; VI: ventilatory index; RSV: respiratory syncitial virus; BPD: broncho-pulmonary dysplasia.

**Figure 1:**
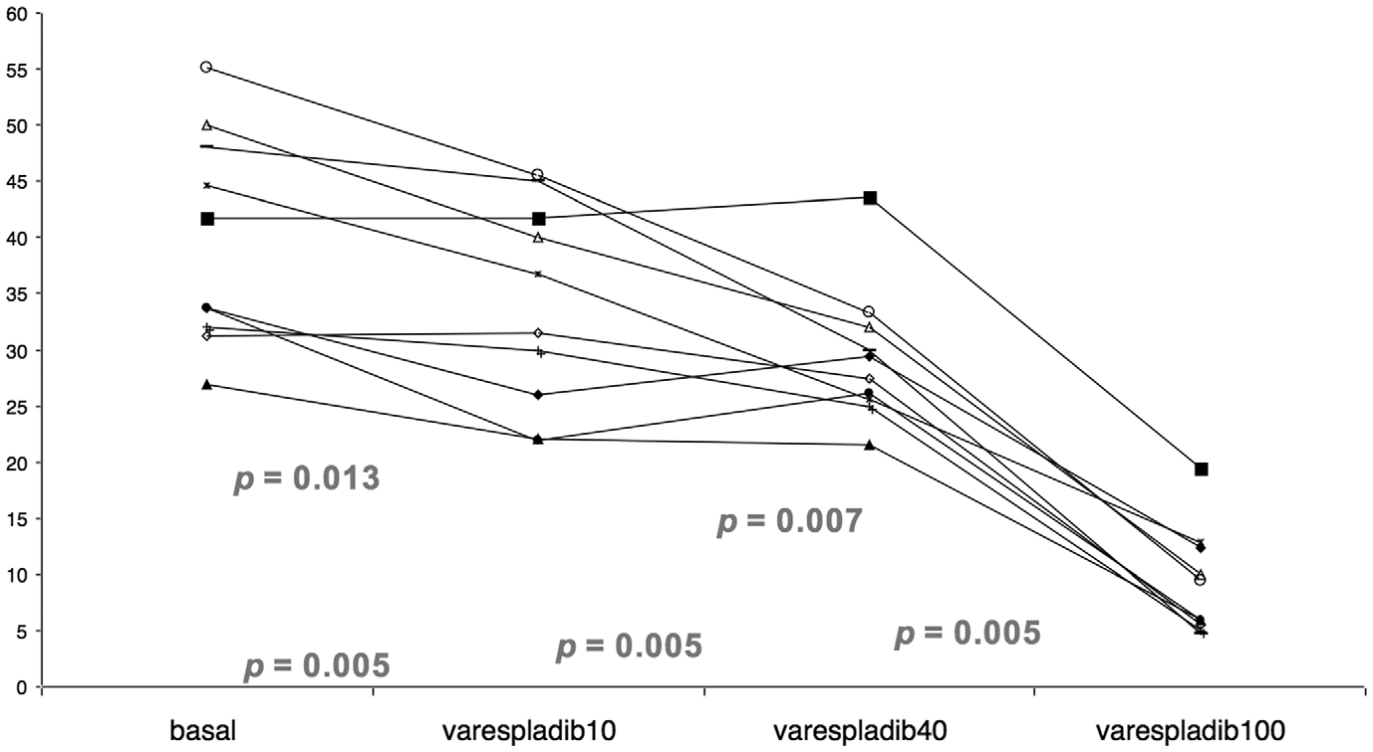
sPLA2 activity in the four BAL aliquots treated either with normal saline (basal), or varespladib at 10, at 40 and at 100 µM. Overall difference in median sPLA2 activity is significant at the Friedman *Q*-test (see text for details). *p*-values describe *post-hoc* comparison: *p* = 0.013 between basal and varespladib10; *p* = 0.007 between basal and varespladib40; *p* = 0.005 between varespladib100 and each other measurement. sPLA2: secretory phospholipase A2.

sPLA2 activity in all BAL aliquots, added with either saline or increasing varespladib concentrations, are depicted in Fig. 1. An overall significant difference (*p*<0.0001) exists among sPLA2 activity in basal conditions [37.7 (31.8–48.5) IU/mL], after addition of 10 µM [34.1 (24.9–42.5) IU/mL], 40 µM (28.4 (25.5–32.3) IU/mL] and 100 µM of varespladib [7.7 (5.4–12.5) IU/mL]. Significant differences are evident at the *post-hoc* comparisons, except that between enzymatic levels measured with 10 and 40 µM of varespladib.

**Figure 2:**
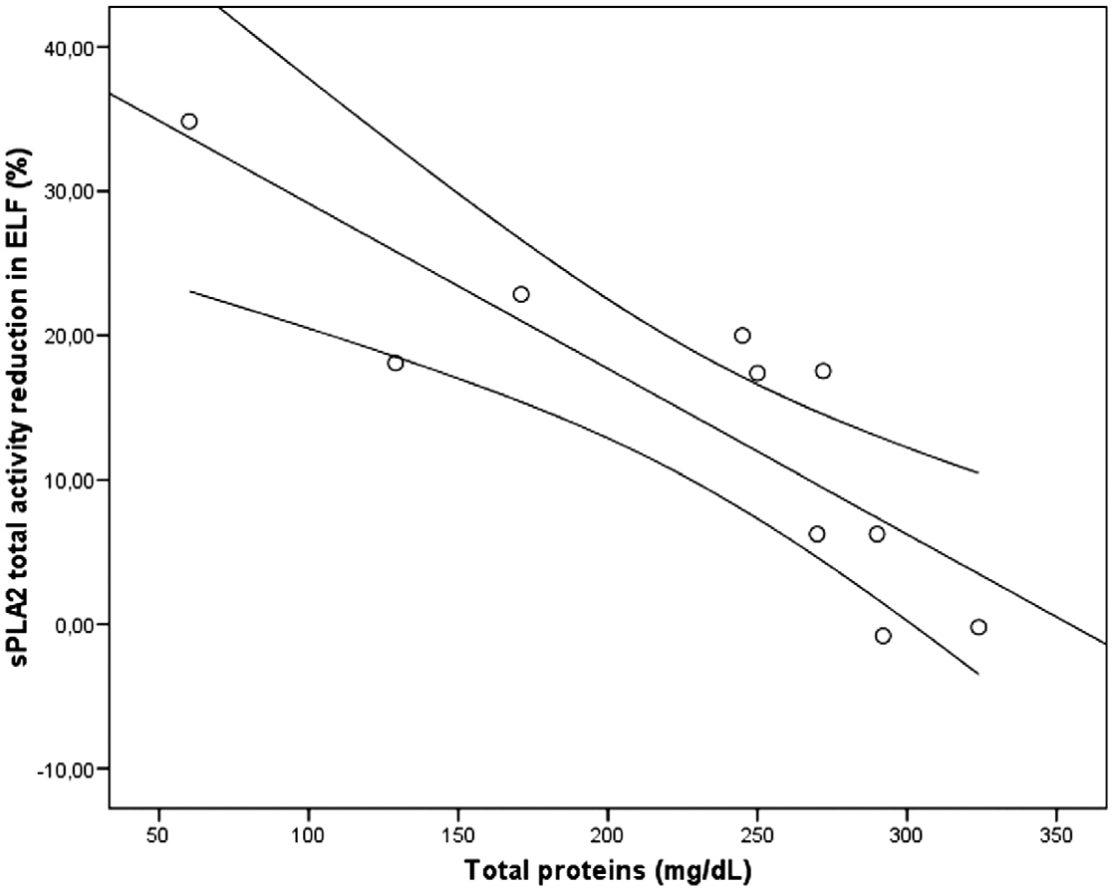
Negative correlation between the reduction of sPLA2 total activity and protein content. Grey lines represent linear regression line and its 95% confidence interval (rho = 0.906; *p*<0.001; R^2^ = 0.73).

Inverse significant correlation was found between protein level and sPLA2 activity reduction at 10 µM of varespladib (rho = −0.906; see Fig. 2). Conversely, no significant relationships were found at 40 (rho = −0.25; *p* = 0.49) and 100 µM of varespladib (rho = −0.05; *p* = 0.88).

Correlations were also found between sPLA2 activity reduction-to-ELF proteins ratio and VI (rho = −0.4; *p* = 0.038), OI (rho = −0.6; *p*<0.001), PaO_2_/FiO_2_ ratio (rho = 0.7;*p*<0.001) and PaO_2_ (rho = 0.63; *p*<0.001). No other correlations were found.

**Figure 3:**
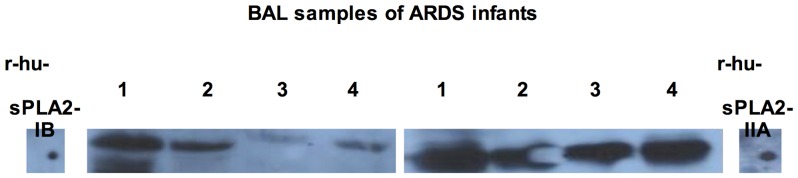
Western blot assay of BAL samples for the two main sPLA2 isotypes that respond differently to varespladib inhibition (sPLA2-IB and sPLA2-IIA). Illustrative results of four ARDS patients (and human recombinant proteins, as controls) are shown. All patients presents sPLA2-IIA, while some also shown the presence of the isotype –IB. sPLA2: secretory phospholipase A2.

Figure 3 shows the western blotting assay of BAL samples for four patients. A relevant amount of sPLA2-IIA was present in all patients, while some also express a lower amount of the isotype –IB.

## Discussion

Varespladib significantly inhibits sPLA2 in BAL of infants affected by post-neonatal ARDS and the inhibition seems to be inversely related to the severity of gas exchange impairment. The present findings seem consistent with another study [9] reporting significant correlations between sPLA2, the degree of ventilatory support and the gas exchange impairment. These findings, though intriguing, are not describing the clinical administration of varespladib to patients. In fact, the interaction with cells and whole tissues may influence the effect of varespladib and, consequently, the surfactant function. However, these findings allow to speculate on interesting application of varespladib and future research steps.

When administered intravenously in rabbit acute lung injury, varespladib was able to improve gas exchange, compliance and reduce inflammatory mediators [5], [6]. Recently, we were able to report a similar effect of varespladib in some types of neonatal lung injury, such as hyaline membrane disease or meconium aspiration syndrome, that are very different from ARDS [28], [29]. We now add novel findings for this particular field: in fact, paediatric ARDS is well distinct from neonatal lung injuries. It shares some features with the adult form of the syndrome but differ in mortality, triggers and epidemiology, needing specific paediatric investigation [29].

*Ex vivo* IC_50_ of varespladib in BAL from ARDS infants is roughly well over the nanomolar range described in *other* experiments and this may be explained for three reasons [13]. First, high protein levels are present in the alveoli of ARDS patients and this could reduce the amount of free varespladib. In fact, varespladib binds such proteins and this prevents it from reaching the sPLA2 catalytic site, as it has been shown using animal data [13]. The inverse correlation between sPLA2 activity reduction and protein content is significant only at the lowest varespladib concentrations, while correlation coefficient is reducing with increasing doses. This is consistent with data already published about varespladib-protein binding [13] and suggests that high varespladib dosing may overcome this problem. In fact, animals with lung injury clearly showed a dose-dependent effect [5], [6]. Second, the different proportions of sPLA2 isotypes expressed in these young patients may be relevant: in fact, we clearly show that some patients also have a detectable amount sPLA2-IB, which is less sensible to varespladib inhibition [13]. Third, different type and amount of surfactant phospholipids present in the diseased alveoli may also play a role [30], [31]. Total phospholipid content in our population is low and consistent with other ARDS patients [31] albeit qualitative alteration of phospholipid pool may deserve to be studied. Clearly, further research is needed to clarify the main factor affecting the varespladib inhibition.

sPLA2 seems to be less inhibited in samples associated with severe gas exchange impairment. OI, PaO_2_/FiO_2_ ratio and VI describe both oxygenation and ventilation: both components of gas exchange are impaired and inversely correlated with the sPLA2 activity reduction/proteins ratio. This phenomenon could be related to a higher enzyme production or to a change in the expression profile of different isotypes requiring much varespladib to be inhibited. Alternatively, a greater degree of inflammation and more proteins filling the alveoli could interfere with the enzymatic inhibition. Finally, the onset of a fibro-proliferative process could influence the inhibition. sPLA2 correlates with lung stiffness [7], which in its turn is associated with collagen activation and deposition [8]. This fibrotic process is known to correlate negatively with clinical outcome [32], [33] and, in fact, dying patients have higher alveolar sPLA2 levels [4].

Some particular characteristics of our population should be noticed. First, patients were mostly affected by quite severe forms of ARDS (as shown by the low phospholipid content, the severe PaO_2_/FiO_2_ ratio, OI, VI, Murray's score and the long duration of mechanical ventilation). Actually, ARDS is a complex syndrome with many possible causes and these may affect the permeability of the alveolar/blood barrier causing various degree of lung oedema [4], [9]. The different causes of ARDS may have influenced our findings. For instance, three of our cases were induced by RSV infection. RSV, the commonest cause of viral respiratory infections in infants [34], triggers an enzymatic cross-talk inducing the over-expression of the cytosolic phospholipase A2 [35], which in its turn may boost the production of sPLA2, both directly and through its inflammatory products [36], [37].

Varespladib is clinically interesting because it is a sPLA2 inhibitor with wide existing pharmacological data, having already undertaken phase II/III clinical studies in adults (in both the oral and intravenous formulations), for different clinical indications [15]–[19]. Such drug might achieve the double goal of: 1) reducing lung tissue inflammation, and 2) sparing the endogenous pool of surfactant. This is an unique key point in acute lung injury and ARDS, because both processes (inflammation and surfactant catabolism) are linked by sPLA2 [10]. Such link is confirmed by the lower levels of Clara Cell Secretory Protein, the natural sPLA2 inhibitor, found in BAL of adults with ARDS [38]. Varespladib could surrogate locally this natural inhibition, while other therapeutics fail. In fact, steroid treatment has not definite advantages in adults with ARDS [39] and may have significant side effects in small infants; moreover, in rabbits, steroids effectively reduce inflammation, but fail to reduce sPLA2 activity and to protect surfactant [40].

We acknowledge some study limitations. Ours is a preliminary experience on a small population, sampled only once at the diagnosis of ARDS. However, our population size is similar to the studies performed in this field in adults [3], [4] and we provide definite data from our monitoring system to study the relationships with clinical variables. Larger studies including ARDS induced by several causes should verify if the underlying diseases, type of ARDS or other clinical characteristics may influence the inhibition of sPLA2. Similarly, the whole sPLA2 pathway, profile of expression of enzyme isotypes, role of different phospholipids and impending fibro-proliferative process deserve to be studied. Our study is based on a cell-free model, therefore no cellular interactions may be taken into account. In conclusion, varespladib may significantly inhibit sPLA2, providing efficacious enzyme- and tissue-specific anti-inflammatory action at lung level. Specific studies should address the above described issues in animal models, to define the main factors affecting sPLA2 inhibition and to also investigate the best timing and dosing of administration.
